# Evaluating a Prototype Nasolaryngoscopy Hood During Aerosol-Generating Procedures in Otolaryngology

**DOI:** 10.1177/0194599820973652

**Published:** 2020-11-24

**Authors:** Michal J. Plocienniczak, Ravi Patel, Jessica Pisegna, Gregory Grillone, Christopher D. Brook

**Affiliations:** 1 School of Medicine, Boston University, Boston, Massachusetts, USA; 2 Department of Otolaryngology–Head and Neck Surgery, Boston Medical Center, Boston, Massachusetts, USA

**Keywords:** aerosol, COVID-19, otolaryngology, endoscopy, prevention

## Abstract

**Objective:**

During the COVID-19 pandemic, there has been considerable interest in identifying aerosol- and droplet-generating procedures, as well as efforts to mitigate the spread of these potentially dangerous particulates. This study evaluated the efficacy of a prototype nasolaryngoscopy hood (PNLH) during various clinical scenarios that are known to generate aerosols and droplets.

**Study Design:**

Prospective detection of airborne aerosol generation during clinical simulation while wearing an PNLH.

**Setting:**

Clinical examination room.

**Methods:**

A particle counter was used to calculate the average number of 0.3-µm particles/L detected during various clinical scenarios that included sneezing, nasolaryngoscopy, sneezing during nasolaryngoscopy, and topical spray administration. Experiments were repeated to compare the PNLH versus no protection. During the sneeze experiments, additional measurements with a conventional N95 were documented.

**Results:**

There was a significant increase in aerosols detected during sneezing, sneezing during nasolaryngoscopy, and spray administration, as compared with baseline when no patient barrier was used. With the PNLH in place, the level of aerosols returned to comparable baseline levels in each scenario. Of note, routine nasolaryngoscopy did not lead to a statistically significant increase in aerosols.

**Conclusion:**

This study demonstrated that the PNLH is a safe and effective form of protection that can be used in clinical practice to help mitigate the generation of aerosols during nasolaryngoscopy. While nasolaryngoscopy itself was not shown to produce significant aerosols, the PNLH managed to lessen the aerosol burden during sneezing episodes associated with nasolaryngoscopy.

The COVID-19 pandemic has continued to cause dramatic shifts in the practice of otolaryngology. In the early phases of the pandemic, studies demonstrated the particular risks that otolaryngologists face during routine practice.^1,2^ Studies suggested the ability for viral particles to be exhaled in breaths and coughs in pediatric and adult patients in the form of aerosols.^3-6^ Even with standard precautions such as physical distancing and face masks, aerosols pose a risk.^2,7^ Aerosol-generating procedures such as nasolaryngoscopy and intranasal instrumentation were determined to carry a risk of potential transmission if not adequately protected.^8-13^

At the time of this writing, as the number of cases plateau in certain parts of the country, more otolaryngology clinics are reopening for face-to-face appointments. As a result, there has been a strong interest in mitigating the effects of aerosol- and droplet-generating procedures for the safety of patients as well as providers. Certain clinical activities, such as nasolaryngoscopy, administration of topical lidocaine and decongestant sprays, speech, and sneezing, have been shown to generate airborne particulate matter with possible associated risks for transmission.^10,14^ Most notably, when compared with N95 masks, standard surgical masks have been shown to be inadequate at mitigating particulate spread.^2,14^ Modifying N95 masks by creating special holes for nasolaryngoscopy was proposed as a safe solution to protect patients and providers.^14^ However, such modifications can create challenges in performing safe and comfortable nasolaryngoscopy without adequate visualization and flexibility. A more effective method to protect providers and patients during high-risk routine otolaryngologic procedures in a safe and comfortable manner for patients is still needed.

The purpose of this study was to develop and evaluate the efficacy of a prototype nasolaryngoscopy hood (PNLH) that can be worn by the patient to protect the provider against significant aerosol and droplet spread during nasolaryngoscopy. The PNLH was tested during various clinical scenarios, such as sneezing and lidocaine spray administration, in which significant aerosol generation is typically noted.^11,12^ We hypothesize that the PNLH will significantly reduce aerosol generation and safely mitigate the risks associated with such procedures. A separate aim of this project was to provide additional data on aerosol generation during these high-risk procedures.

## Materials and Methods

### Study Design

All experiments in this study were performed on 1 volunteer in a dedicated clinic examination room (80 sq ft) equipped with standard hospital ventilation systems that exchanged the air on average 6 times each hour. The door to the clinic room was kept closed during the experiments, and the number of people and their movement inside the room were kept to a minimum and not changed throughout the protocols. After review by the Boston Medical Center and Boston University Medical Campus Institutional Review Board, this study qualified as “not human subjects research” based on the definitions of human subject and research under the policies and procedures of the Human Research Protection Program, and it was exempt from further review.

### Equipment

**Figure 1.**
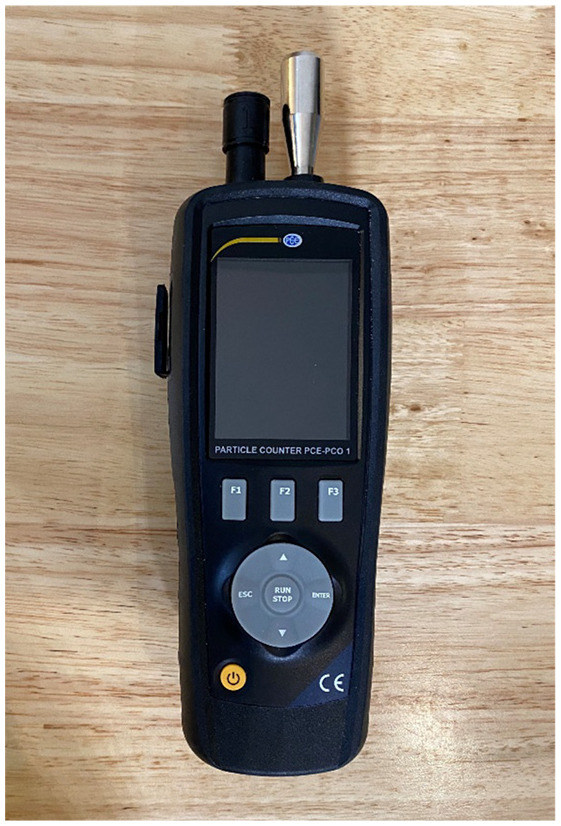
PCE-PCO 1 particle counter (PCE Instruments).

**Figure 2.**
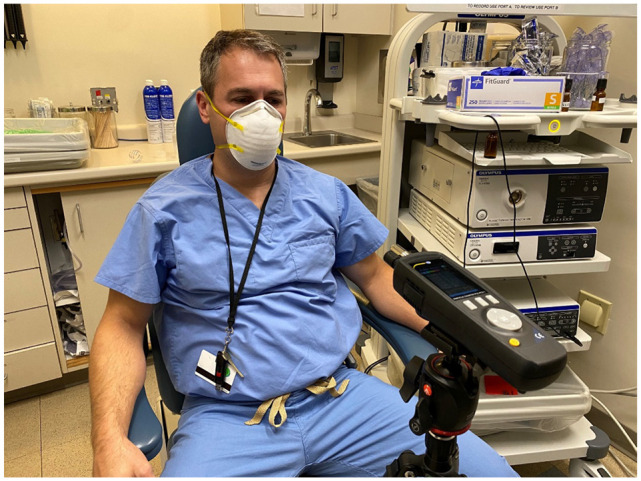
Participant sitting upright in clinical examination room chair 16 in from the intake port on particle counter.

To quantify the aerosols, a PCE-PCO 1 particle counter (**Figure 1**) was used (PCE Instruments).^15^ This device can detect particles from 0.3 to 25 µm, and it achieves this by utilizing a laser and optical sensor to count how many particles are collected inside by an internal pump. Each measurement lasted 21 seconds, which is equivalent to 1 L of air sampled and delivered into the device. For each condition tested, the counter was placed on a tripod at mouth level of the seated participant (55 cm off the ground) and 16 in (approximately 40 cm) in front of the mouth to resemble a realistic distance between provider and patient (**Figure 2**).

**Figure 3.**
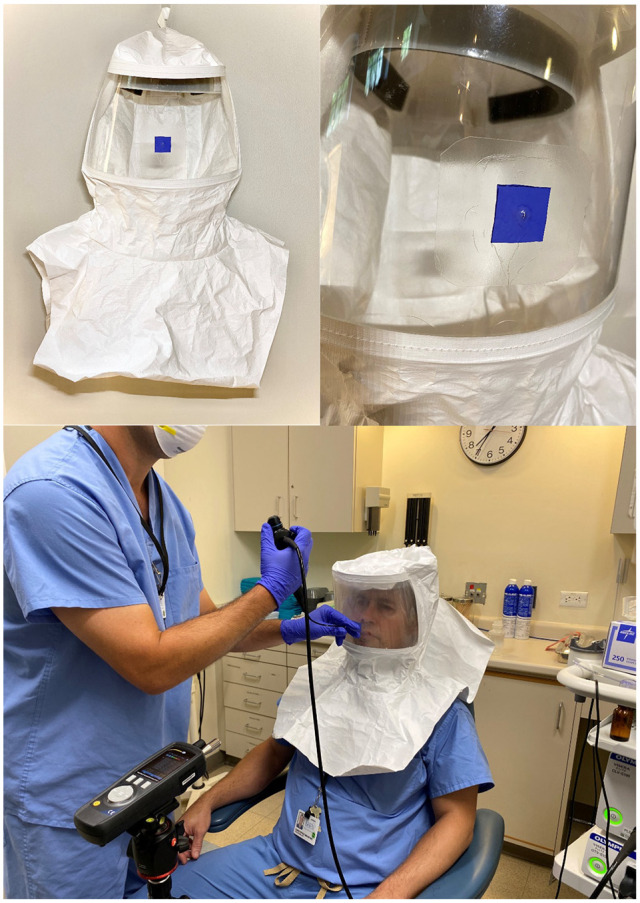
*Upper*: Modified hood with 1-cm hole covered by a patch from a nitrile glove, adhered with Tegaderm. *Lower*: Nasal flexible laryngoscopy through the port constructed on the prototype nasolaryngoscopy hood (PNLH).

The PNLH that was tested in this study was constructed by modifying a Tyvek Supplied Air Respirator Hood (Allergo Industries). A 1-cm hole was drilled into the front of the clear plastic at the location where the flexible nasal endoscope would be introduced. A square piece of rubber (2.5 × 2.5 cm) was cut from a nonsterile glove and placed directly over the drilled hole and secured in place by a Tegaderm patch (6 × 7 cm). This design allowed the clinician to cut a 3-mm hole into the glove immediately before use to insert a lubricated flexible nasal endoscope through the port while maintaining a tight seal around it (**Figure 3**).

### Clinical Simulation

**Figure 4.**
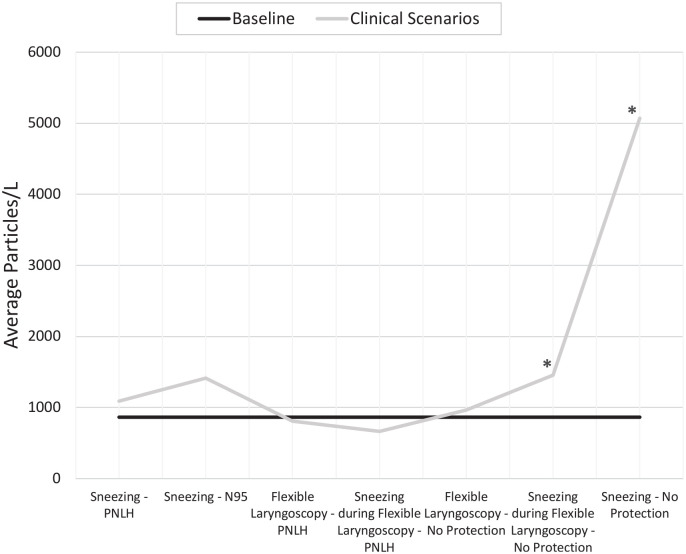
Measuring average particles per liter during different clinical activities. PNLH, prototype nasolaryngoscopy hood. *Statistically significant difference from baseline.

The volunteer was seated upright in a clinical examination room chair with the mouth 16 in from the intake port on the particle counter. This was chosen to approximate the distance from a patient to an endoscopist. Each sample measurement lasted 21 seconds, beginning with baseline samples taken to measure the background particulate matter in the room before any conditions were tested. Time was taken between testing conditions to allow the particle count to return to baseline levels. Each condition was repeated with 4 or 8 samples per condition. The conditions tested, in order, included (1) sneezing with the PNLH on the participant, (2) sneezing with a N95 on the participant, (3) nasolaryngoscopy through the PNLH (**Figure 3**), (4) simultaneous nasolaryngoscopy and sneezing through the PNLH, (5) nasolaryngoscopy with no protection, (6) simultaneous nasolaryngoscopy and sneezing with no protection, and (7) sneezing without protection. For all conditions involving simulated sneezing, 3 separate sneezes were performed per 21-second sample interval. The volunteer forcefully sneezed with the same effort and number of attempts per each 21-second sample. Simulated nasolaryngoscopy was done by intranasal placement of a flexible laryngoscope and advanced to the level of the vocal cords. For conditions involving the PNLH, the flexible laryngoscope was inserted through the constructed port in the front of the hood.

Four additional conditions were tested with an atomizer spray bottle containing saline to simulate lidocaine spray administration. When pumped, the spray bottle released a fine mist amounting to 0.12 mL of liquid, and this was done once per 21-second sample. In all conditions, the nozzle was introduced under the hood and advanced carefully up toward the participant’s face. In terms of positioning, the nozzle was pointed vertically (ie, directly at the ceiling) and maintained 16 in away from the intake port on the particle counter, which was outside the hood. Four samples were taken for each condition. The conditions tested included (1) spray within PNLH, (2) spray within the PNLH with the endoscope in place, and (3) spray with no protection.

### Statistics

The average number of 0.3-µm particles detected was calculated during the various clinical scenarios as well as baseline measurements. The averages were compared with 2-sample *t* tests. To account for the 7 scenarios that were being tested during nasolaryngoscopy and sneezing, a Bonferroni correction was used to adjust statistical significance when *P* < .007. For the series of 3 experiments involving topical lidocaine spray, a Bonferroni-corrected *P* < .017 indicated statistical significance.

## Results

### Sneezing

For the clinical scenarios involving sneezing, 51 measurements were taken on the particle counter throughout the experiments. The 0.3-µm particle counts were documented. Prior to each scenario, baseline measurements were made and averaged. This average baseline was compared with each clinical scenario. The average baseline level was 864 particles/L. When the participant sneezed with no protection, there was a significant increase in particles detected, at 5070 particles/L (*P* < .001). When the volunteer wore the PNLH, the average particles detected totaled 1090 particles/L, which was not significantly greater than baseline (*P* = .132). Finally, when the participant wore an N95 mask, there was an increase in particles detected as compared with baseline, at 1412 particles/L, but this did not reach corrected statistical significance (*P* = .01) (**Figure 4**).

### Endoscopy

For the clinical scenarios involving flexible nasolaryngoscopy, 49 measurements were taken with the particle counter throughout the experiment, again set to 0.3 µm. The same baseline levels were obtained. During flexible nasolaryngoscopy, 963 particles/L were detected on average, which was not statistically different from baseline (*P* = .387). With the PNLH on, the number of particles trended lower to levels comparable to baseline, at 810 particles/L (*P* = .191). However, when the participant sneezed during nasolaryngoscopy, there was a statistically significant increase in particles detected as compared with baseline, for an average of 1394 particles/L (*P* = .001). With the PNLH on, there was a decrease in aerosols detected to levels below baseline during concurrent nasolaryngoscopy and sneezing (**Figure 4**).

### Topical Lidocaine Spray Administration

**Figure 5.**
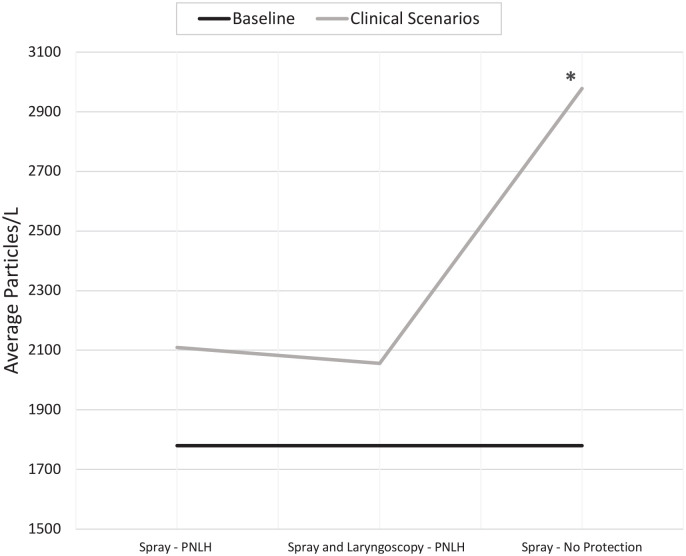
Measuring average particles per liter during different clinical activities involving administration of topical spray. PNLH, prototype nasolaryngoscopy hood. *Statistically significant difference from baseline.

A total of 36 measurements were taken during the clinical scenario involving spray administration with the particle meter set to 0.3 µm. The particle meter was recalibrated, and the baseline was again obtained. When compared with an average new baseline level of 1780 particles/L, there was a statistically significant increase in particles detected during spray administration without any protection, at 2978 particles/L (*P* = .002). With the PNLH in place, there was a marked decrease in particles detected, at 2190 particles/L, which was no longer significantly greater than baseline (*P* = .37). A similar result was achieved when spray was administered with the endoscope placed through the PNLH, with an average 2046 particles/L detected, which was also not significantly different from baseline (*P* = .41; **Figure 5**).

## Discussion

There has been an increased focus in research efforts toward safe otolaryngology practices in the setting of the COVID-19 pandemic. In light of the particular risks that otolaryngologists face during certain aerosol- and droplet-generating procedures, such as nasolaryngoscopy, there is a demand for developing simple, cost-effective, and protective solutions that help mitigate the spread of such particulates and thus protect both patient and physician. Here we present a novel device, the PNLH, that patients can wear comfortably, that allows physicians to perform safe routine nasolaryngoscopy (**Figure 3**), and that has been demonstrated to reduce the physician’s exposure to aerosols.

In an effort to identify high-risk aerosol- and droplet-generating procedures as well as techniques to mitigate these risks, Bleier et al measured particle production during various clinical and surgical scenarios.^14^ During simulated clinical activity, airborne aerosol was detected during nasolaryngoscopy, speech, and sneezing. To lessen the risk, intact or VENT-modified (valved endoscopy of the nose and throat) N95 respirators were used to significantly decrease airborne aerosol transmission. Our findings support some of the conclusions reached by Bleier et al, particularly in regard to sneezing. Our results also demonstrated a significant increase in particle detection as compared with baseline during sneezing. In regard to mitigating aerosol spread during sneezing, our results suggest that the N95 mask as well as the PNLH significantly reduced aerosols detected. However, wearing an N95 resulted in 30% more aerosols being detected as compared with the PNLH, although this did not reach the corrected statistical significance (*P* = .01). Clinically, our findings suggest that the PNLH is likely a more effective barrier protecting endoscopists from a patient’s sneeze.

With regard to nasolaryngoscopy, there was a slight increase in aerosols detected in our experiments, but this did not reach statistical significance when compared with baseline (*P* = .39). This differs from the study by Bleier et al. A possible explanation for this difference is that our particle counter was placed at a distance of 16 in (~40 cm) rather than 15 cm. Our goal was to ensure that the aerosols being measured were at a distance most likely to resemble that between patients and otolaryngologists during nasolaryngoscopy. However, in a more recent study, Rameau et al evaluated aerosol-generating procedures and determined that flexible nasolaryngoscopy does not generate significant aerosols, even with the particle counter placed 12 cm from the participant’s oral cavity.^16^ Our work demonstrated a significant increase in aerosols when the volunteer sneezed during flexible endoscopy. To mitigate this risk, the PNLH was again tested in this particular clinical scenario, and it showed a reduction in particle detection to levels below baseline.

With regard to topical spray administration, this study showed a statistically significant increase in particles detected against baseline (*P* < .05). To mitigate the spread, the PNLH was again placed on the participant and the spray administered. Our results show that the PNLH provides an effective means of mitigating the spread of aerosols, even during a highly aerosolizing procedure such as spray administration, with significant reductions in particles detected to levels comparable to baseline (*P* = .37). Even with an endoscope in place through the PNLH, the levels of aerosol did not differ significantly from baseline after spray administration (*P* = .41).

This study evaluated the efficacy of a prototype device, the PNLH, during various common otolaryngologic procedures that have been shown to generate significant aerosols and droplets and therefore pose a risk to providers. With this simple intervention, the data support our hypothesis, and patients can comfortably wear the PNLH and undergo routine flexible nasal laryngoscopy, even with topical lidocaine spray administration, with no risk to the provider. If patients begin to sneeze during the examination, our data suggest that providers will remain protected through the use of the PNLH. Finally, because of the clear visor protecting patients, we noted that providers performing flexible laryngoscopy during these experiments felt much more comfortable initially advancing the scope toward the nasal cavity, since the patient’s face was visible the entire time. Once it was within the nasal cavity, providers noted persistent ease of performing nasolaryngoscopy, as the PNLH served as extra support while the scope was advanced.

While this study evaluated the efficacy of the PNLH in the setting of a routine nasolaryngoscopy in reducing aerosol spread, there are other possible applications but also one major barrier to overcome. For the series of experiments featured in this study, only 1 PNLH was constructed and used on 1 test participant. In the prototype model, the Tegaderm and rubber glove piece are easily replaceable, which could facilitate a disinfection protocol in the future. For clinical use, either the PNLH would need to be mass produced to allow for single use, or a safe and effective protocol of cleaning and reusing each PNLH would need to be established. Furthermore, only 1 size of the PNLH was tested, which may not fit every patient with various head shapes and hair styles.

There are several limitations to this study. First, the particle counter measured particle sizes averaging 0.3 µm and not the presence of aerosolized viral nuclei, which can be <0.1 µm.^17^ Second, baseline levels of aerosol changed throughout the experiments, with marked increases in baseline aerosol during the latter half of the experiments, which focused on nasal sprays. While the particle counter was adjusted and calibrated prior to each experiment, it is important to note the variability of background aerosols and their potential effect on the readings in real time. Finally, a single participant was asked to simulate sneezes during the data collection phase. While the volunteer remained consistent with 3 sneezes per 21-second sample and attempted to keep the force of sneezes consistent, there is obviously variability of force and aerosol generation and the possibility of fatigue over the course of the experiment.

Thus far, the PNLH has been limited to nasolaryngoscopy. Perhaps in the near future, similar experiments can be repeated with the presence of 2 holes fashioned in a similar manner to allow not only flexible nasolaryngoscopy but an additional port for instrumentation and possible interventions.

## Conclusion

Efforts to lessen aerosol and droplet spread during routine otolaryngologic physical examinations are underway in the setting of the COVID-19 pandemic. Here we present a prototype barrier, the PNLH, that has proven to be an effective method of reducing the spread of aerosols and droplets. The most significant generators of aerosol in this study were sneezing, sneezing during nasolaryngoscopy, and topical lidocaine spray administration, all of which were successfully mitigated through the use of the PNLH.
